# Influence of Onion Peel Extract on the Dough Characteristics of High-Gluten Wheat Flour and the Quality of Bread

**DOI:** 10.3390/foods14091618

**Published:** 2025-05-03

**Authors:** Cuntang Wang, Yuqing Wang, Ning Wang, Jian Ren

**Affiliations:** 1 College of Food and Bioengineering, Qiqihar University, Qiqihar 161006, China; 2 Engineering Research Center of Plant Food Processing Technology, Ministry of Education of China, Qiqihar 161006, China

**Keywords:** bread, dough, onion peel extract, antioxidant, high-gluten wheat flour

## Abstract

In this study, we evaluated the effect of onion peel extract (OPE), which is rich in phenolics and flavonoids, on the performance of high-gluten wheat flour and bread quality to meet consumer demand for functional bakery products. The addition levels of OPE were set at 0%, 0.25%, 0.5%, 0.75% and 1% (*w*/*w*), respectively, to analyze their effects on water/oil absorption capacity, falling number, and rheological properties (farinographic properties, tensile properties, dynamic rheological properties and gelatinization characteristics) of dough, as well as bread quality (antioxidant activity, texture, microstructure, specific volume and sensory evaluation). When the OPE addition level was 0.25% and 0.5%, the dough’s oil absorption capacity, farinographic properties, tensile properties, dynamic rheological properties, and gelatinization characteristics were all improved. Correspondingly, a more compact and ordered microstructure was observed in the dough. It was found that total phenolic content, total flavonoid content, and antioxidant capacity of bread significantly increased with the increase in the OPE addition level (*p* < 0.05). The texture analysis of the bread showed that the addition of OPE reduces the hardness and chewiness of bread, indicating that the texture of bread was easily accepted by consumers. In the sensory evaluation, when the addition level of OPE was 0.5%, the color and flavor of bread were improved, and the overall acceptability was relatively high. In conclusion, OPE has improved the texture characteristics and nutritional value of bread. It is recommended that the addition level of OPE in high-gluten wheat flour dough and bread be below 0.5%.

## 1. Introduction

Consumer expectations for food goods have changed as a result of the growing emphasis on health around the world. Foods that provide both nutritional advantages and sensory pleasure are becoming more and more popular, rather than only being a source of satiety [1]. Human nutrition depends heavily on flour, especially high-gluten wheat flour, which is an essential component of bread. In recent years, the global baking food business has grown significantly. It is expected to reach a scale of 485 billion US dollars by 2025 [2]. When high-gluten wheat flour is ground, the bran and germ are removed. This will lead to the loss of 60–80% of important bioactive substances in wheat products, such as flavonoids and total phenolic compounds [3]. Therefore, food scientists and researchers are increasingly concentrating on improving the nutritional value of high-gluten wheat flour and its products by adding functional ingredients [4]. Many nations have enacted laws requiring the fortification of high-gluten wheat flour with minerals including iron and zinc, ascorbic acid, folic acid, and B vitamins in order to make up for these nutritional losses [5]. Although they have certain antioxidant advantages, their bioavailability, functional mechanisms, and long-term safety are quite different from those of the naturally occurring bioactive chemicals in plants [6]. Therefore, a major trend in the food processing industry is to incorporate natural antioxidants into daily staple foods. In addition to enhancing the antioxidant capacity of these foods, natural antioxidants can also extend product shelf life, improve product texture, and enhance overall sensory appeal. [7]. Extracting antioxidant compounds from plant by-products has recently garnered significant attention for enhancing resource efficiency and food value.

As one of the most widely cultivated horticultural crops, onion (*Allium cepa* L.) has a global planting area of approximately 3.7 million hectares and yields up to 100 million tons annually [8]. The United States, Egypt, Turkey, India, and China collectively produce 80% of the world’s onions. Onion peel, leaves, and underdeveloped or sick onions are among the 100 million tons of waste that comes from the production and processing of onions, which is both an environmental issue and causes financial losses [9,10]. Studies have found that onion by-products, particularly the peels and outer scales, are rich in bioactive substances such as phenolics, flavonoids, quercetin, anthocyanins, vanillic acid, and ferulic acid [11].

The structure and texture of bread depend on the gluten network that is created by the proteins in high-gluten wheat flour. To provide the desired bread volume and texture, a strong gluten network gives the dough the elasticity and gas-holding capacity it needs [12]. Nevertheless, adding polyphenolic chemicals could disrupt the gluten network and change the characteristics of the dough. Gluten proteins’ amino acids can bind to polyphenols, changing their structure and functionality [13]. These interactions could affect the bread 14’s quality by affecting the dough’s flexibility, tensile strength, and gas-holding capacity [14]. In addition to affecting dough properties, polyphenols can alter the flavor, texture and color of bread, all of which can have a significant impact on consumer acceptance [15]. Additionally, polyphenols’ antioxidant qualities prolong the bread’s shelf life by preventing lipid oxidation during storage [16]. Plant polyphenols are a promising component for the creation of functional bread because of these sensory enhancements as well as the health advantages of consuming polyphenols. Previous studies have applied plant-derived extracts in bread, including carob extract [15], apple pomace [17], rosehip extract [18], berry extract [19], and water sumac extract [20]. In addition to enhancing their antioxidant activity, these plant polyphenols can also improve the structural and sensory qualities of bread. Plant polyphenols have many benefits, but incorporating them into the bread-making process necessitates careful optimization to balance their impact on dough properties and sensory qualities. While low concentrations may increase the bread’s antioxidant capacity and quality, high quantities of plant polyphenols may damage the gluten network and impair the bread’s texture and specific volume [21]. Therefore, figuring out the ideal polyphenol concentration is essential to optimizing their advantages without sacrificing bread quality.

In conclusion, onion peel extract as a potential natural antioxidant has broad application prospects in improving the quality of high-gluten flour and its products. However, there are still many deficiencies in the current systematic studies on the rheological properties and microstructure of high-gluten wheat flour dough extracted from onion peel extract, as well as the effects on the texture, antioxidant activity and sensory properties of bread. This study aims to systematically investigate the effects of onion peel extract as a functional component on the above characteristics of high-gluten wheat flour, reveal its mechanism of action, provide theoretical basis and technical support for the rational application of onion peel extract in food industry, and also provide a new method for the quality improvement and functional improvement of high-gluten wheat flour products.

## 2. Materials and Methods

High-gluten wheat flour (without any additives) was purchased from the local supermarket. The yellow onion peel was provided by the farmers’ market in Qiqihar, China (without any signs of mold infection). Prior to extraction, the yellow onion peel was thoroughly washed to remove surface dirt, debris, and other contaminants then dried in an electric blast-drying oven (DAF-4A, Feisifu Biotechnology Co., Ltd., Haikou, China) at 40 °C. After drying, the material was ground using a crusher (GX-220, Zhejiang Gaoxin Industry and Trade Co., Ltd., Jinhua, China) and passed through a 100-mesh sieve for further use. Sodium nitrite, sodium hydroxide, sodium carbonate, aluminum chloride, quercetin, Folin-Ciocalteu reagent, gallic acid, DPPH (2,2-diphenyl-1-picrylhydrazyl), potassium persulfate, ABTS (2,2′-azino-bis-3-ethylbenzothiazoline-6-sulphonic acid), and other chemicals were purchased from Shanghai Eon Chemical Technology Co., Ltd., Shanghai, China.

### 2.1. Preparation of the Onion Peel Extract

The preparation of onion peel extract (OPE) was carried out according to the method of Da et al. [22] with slight modifications. The entire process consisted of two extraction cycles per sample (first extraction of 50 g of powder with 500 mL of 70% ethanol, followed by filtration, centrifugation, and collection of the supernatant). After mixing the supernatants from two cycles of each experiment, the extracts were concentrated by vacuum evaporation at 50 °C to remove ethanol, and the resulting aqueous concentrate was lyophilized (freeze-dried) without additional solvents to obtain the final powder. The procedure was repeated until enough OPE was obtained to conduct the experiments.

### 2.2. Quality Determination of High-Gluten Wheat Flour with Different Levels of OPE

#### 2.2.1. Determination of Water Absorption Capacity and Oil Absorption Capacity

The water absorption capacity (WAC) and oil absorption capacity (OAC) of high-gluten wheat flour with different levels of OPE were evaluated following the procedures outlined by Okwunodulu et al. [23]. For detailed steps, please refer to 2.2.1 of the Supplementary Documents.

#### 2.2.2. Determination of Falling Number

The falling number of high-gluten wheat flour with different levels of OPE was measured using a falling number tester (FN-II, Hangzhou Daji Photoelectric Instrument Co., Ltd., Hangzhou, China), as described by Graybosch et al. [24]. For detailed steps, please refer to 2.2.2 of the Supplementary Documents.

#### 2.2.3. Determination of Farinographic Properties

The farinographic properties of high-gluten wheat flour with different levels of OPE were determined according to Zhang et al. [21] using a farinograph (JFZD300, Beijing Dongfu Jiuheng Instrument Technology Co., Ltd., Beijing, China). The analysis included water absorption, development time, stability time, degree of softening, and farinograph index, as supported by the instrument’s software. For detailed steps, please refer to 2.2.3 of the Supplementary Documents.

#### 2.2.4. Determination of Gelatinization Characteristics

The gelatinization characteristics of high-gluten wheat flour with different levels of OPE were determined using a rapid viscosity analyzer (RVA 4500, Tianjin Fulutong Technology and Trade Co., Ltd., Tianjin, China) according to the method of Wu et al. [25]. Pasting temperature, peak viscosity, breakdown, and setback were analyzed via the instrument’s software. For detailed steps, please refer to 2.2.4 of the Supplementary Documents.

#### 2.2.5. Determination of Tensile Properties

The tensile properties of high-gluten wheat flour with different levels of OPE were evaluated using a tensile tester (JMLD 150, Beijing Dongfu Jiuheng Instrument Technology Co., Ltd., Beijing, China) following the procedure described by Zhang et al. [26]. A 300 g dough sample, prepared with a farinograph (JFZD300, Beijing Dongfu Jiuheng Instrument Technology Co., Ltd., Beijing, China), was molded into a standard cylinder for testing. The dough was subjected to a fermentation process at 30 °C for 45, 90, and 135 min. The dough’s extensibility, tensile resistance, stretch ratio, and stretch curve area were recorded. For detailed steps, please refer to 2.2.5 of the Supplementary Documents.

#### 2.2.6. Determination of Dynamic Rheological Properties

The dynamic rheological properties of high-gluten wheat flour with different levels of OPE were evaluated using a rheometer (KinexusPro+, Malvern Instruments Ltd., Malvern, Worcestershire, UK) following the method outlined by Zhang et al. [21]. The dough was prepared by mixing with a farinograph (JFZD300, Beijing Dongfu Jiuheng Instrument Technology Co., Ltd., Beijing, China) for 5 min. The dough was then allowed to rest at 25 °C for 30 min. The linear viscoelastic region of the samples was determined via strain sweep at 25 °C and 1 Hz. A frequency sweep was subsequently performed to measure the dynamic rheological properties of the dough, using a 40 mm diameter parallel plate, 1.0% strain, 25 °C temperature, a frequency range of 0.1 Hz to 20 Hz, and a 2 mm gap. Finally, the variation of elastic modulus (G′), viscous modulus (G″), and loss factor (tanδ = G″/G′) with frequency was analyzed using rSpace (Version 1.5), the analysis software that comes with the rheometer. 

### 2.3. Bread Quality Analysis

#### 2.3.1. Preparation of Bread

Bread dough was prepared using 120 g of high-gluten wheat flour containing different addition levels of OPE (0.25%, 0.5%, 0.75%, 1%, *w*/*w*), 24 g of sugar, 2 g of yeast, 6.8 g of butter, 1.2 g of salt, and 73 g of water. After mixing the above ingredients, a dough kneader (SM-201/202, Zhejiang Shaoxing SUPOR Household Appliances Co., Ltd., Shaoxing, China) was used to knead the dough into a smooth mass. After the first fermentation in the oven (Midea PT3031, Wuhu Midea Kitchen and Bathroom Appliances Manufacturing Co., Ltd., Wuhu, China) at 35 °C for 20 min, the dough was removed, kneaded, and reshaped again. It was then placed back in the oven for a second fermentation (35 °C, 50 min). After fermentation, the dough was transferred to a baking tray and baked at both upper and lower heat of 175 °C for 20 min. Once baked, the bread was cooled to room temperature and stored in self-sealing bags for subsequent analysis [26].

#### 2.3.2. Determination of Total Phenolic Content, Total Flavonoid Content, and Antioxidant Activity of Crumbs

Preparation of polyphenol extract from crumbs: Crumbs were vacuum freeze-dried for 48 h, then crushed and sieved through a 40-mesh sieve to obtain lyophilized powder. Subsequently, 40 mL of 70% ethanol solution was mixed with 2 g of the lyophilized powder and soaked for 2 h. The mixture was then centrifuged using a centrifuge (TDL-5-A, Beijing Jingli Centrifuge Co., Ltd., Beijing, China) at 3500× *g* rpm for 15 min, and the supernatant was collected for subsequent analysis [27].

The total phenolic content of crumbs was determined following the method outlined by Sagar and Pareek [28]. The results were expressed as milligrams of gallic acid equivalents per gram of dry weight (mg GAE/g DW). For detailed steps, please refer to 2.3.2 of the Supplementary Documents.

Total flavonoid content of crumbs was measured according to the method of Sagar and Pareek [28]. The results were expressed as milligrams of rutin equivalents per gram of dry weight (mg RE/g DW). For detailed steps, please refer to 2.3.2 of the Supplementary Documents.

The DPPH and ABTS free radical scavenging rate were employed to quantify the antioxidant activity of the samples in order to increase its precision and predictability. The DPPH and ABTS radical scavenging rate of crumbs were evaluated using the method described by Sagar and Pareek [22], with slight modifications. For detailed steps, please refer to 2.3.2 of the Supplementary Documents.

#### 2.3.3. Color of Crumbs

The color parameters of the samples, including L* (lightness), a* (red/green), and b* (yellow/blue), were measured using a chromometer (Shenzhen Linshang Technology Co., Ltd., Shenzhen, China), following the methodology outlined by Zhang et al. [21]. These values were obtained based on the CIE (Commission Internationale de l’Éclairage) color scale. The measurement process was performed under standard illuminant conditions, and the observer angle used was 10°, as recommended by the CIE. 

#### 2.3.4. Specific Volume of Bread

According to the method described by Wang et al. [12], the bread’s specific volume was calculated using the millet displacement method. First, a 500 mL graduated cylinder was filled with 500 mL of millet, which was then completely poured into a beaker. To ensure the bottom of the 500 mL graduated cylinder was fully covered, a thin layer of millet was spread at the bottom of the cylinder. The cooled bread was then placed into the cylinder, and millet was added to fill the cylinder up to the 500 mL mark. The remaining millet was measured using a 100 mL graduated cylinder, and this volume was taken as the bread volume. The bread was weighed using an electronic balance to record its mass. The specific volume of bread is defined as the ratio of bread volume to weight (cm^3^/g).

#### 2.3.5. Textural Characteristics of Bread

The measurement of bread texture properties followed the method reported by Wang et al. [12] with minor modifications. Bread samples of 15 mm × 15 mm × 20 mm in size were placed on the platform of a texture analyzer (TA-TX Plus, Stable Micro System, Godalming, Surrey, UK). A 36 mm cylindrical plastic probe was used, with a probe lifting height of 40 mm, a dropping speed of 60 mm/s, and a compression ratio of 50%. The texture parameters measured included hardness (N), cohesiveness, springiness (mm), and chewiness (N).

#### 2.3.6. Microstructure

The procedure for observing the dough microstructure followed the method of Tang et al. [29] with slight modifications. Dough samples were freeze-dried, cut into 2 cm × 2 cm × 2 cm cubes, and fixed with 3% glutaraldehyde. After rinsing with 0.1 mol/L phosphate buffer solution, the samples underwent gradient ethanol dehydration using 30%, 50%, 70%, 90%, and 100% ethanol solutions. Following freeze-drying, the lyophilized samples were attached to a sample stage with conductive adhesive and sputter-coated with gold for 90 s. Microstructural observations were conducted using a cold field emission scanning electron microscope (SEM, Gemini 300, Carl Zeiss AG, Oberkochen, Germany) operated at 20.0 kV. Images of the dough microstructure were captured at 60× magnification to analyze structural changes.

#### 2.3.7. Sensory Evaluation of Bread

The sensory ratings of the samples were evaluated using a nine-point hedonic scale, which includes color, flavor, texture, taste, and overall acceptability (with 1 being severely disliked and 9 being extremely liked), in accordance with the methodology of Sagar and Pareek [28]. The study involved thirty panelists. A random three-digit number was assigned to each sample, which was then arranged on a white plate and presented to the panelists in a random order. To reduce the influence of oral residues on the evaluation, the panelists were instructed to rinse their mouths with warm water before sampling.

### 2.4. Data Analysis

The findings are shown as mean ± standard deviation (SD), and each experiment was run in triplicate. To analyze the data, SPSS software, version 20.0, was used. In this study, the analysis of variances (ANOVA) procedure was used. Additionally, Duncan’s multiple range tests were used to see whether there was a significant difference in the means of the various treatments. Consequently, a statistically significant difference was indicated by *p* < 0.05.

Statement of approval for the experiment: Every experiment was carried out in compliance with the applicable rules and regulations. The academic review commissions of Qiqihar University’s College of Food and Bioengineering approved all experimental protocols prior to analysis.

Ethical statement: Prior to analysis, the academic review commissions of Qiqihar University’s College of Food and Bioengineering examined and authorized the sensory evaluation panelists with written informed permission (Project Number: QH20241210, approved on 10 December 2024).

All participants signed an informed consent form after being fully informed about the study.

## 3. Results and Discussion

### 3.1. Total Phenolic Content, Total Flavonoid Content, and Antioxidant Activity of Onion Peel Extract

Compared to the edible portion of the onion, the outer peel is known to have higher amounts of phenolic chemicals, such as isorhamnetin and quercetin [11]. The onion peel was extracted using an ethanol–solvent technique in this study, and its antioxidant activity, total phenolic content (TPC), and total flavonoid content (TFC) were assessed. The OPE’s TPC and TFC values were determined to be 620.61 mg gallic acid equivalents (GAE)/g DW and 549.36 mg rutin equivalents (RE)/g DW, respectively. Furthermore, the DPPH and ABTS radical scavenging activities were determined to be 90.59 mg ascorbic acid equivalents (AAE)/g DW and 156.17 mg ascorbic acid equivalents (AAE)/g DW, respectively.

Comparing these results with other studies can provide deeper insights into the influencing factors. Singh et al. [30] used a 70% ethanol solution combined with ultrasonic-assisted extraction to isolate bioactive substances from onion peel. Their findings reported that the TPC in onion peel was 418.04 mg GAE/g, TFC was 212.36 mg QE/g, and antioxidant activity was 101.19 mg trolox equivalent/g dry weight. In our study, TPC and TFC were significantly higher than those reported by Singh et al. [30], while the DPPH and ABTS radical scavenging capacities were comparable with the antioxidant activity they described. In addition, Pobiocka et al. [31] extracted flavonoids from different varieties of yellow onion peel using only methanol and obtained lower levels compared to our study, ranging between 2.4 and 12.2 mg quercetin equivalent (QE)/g. Differences in bioactive content and antioxidant activity may be attributed to phytochemical variability in raw materials and differences in solvents and extraction methods used during extraction. Additionally, different sources of raw materials or methods of expressing final results (DW/DOS) can also lead to differences in antioxidant activity [32].

### 3.2. Effect of OPE on WAC and OAC of High-Gluten Wheat Flour

**Figure 1:**
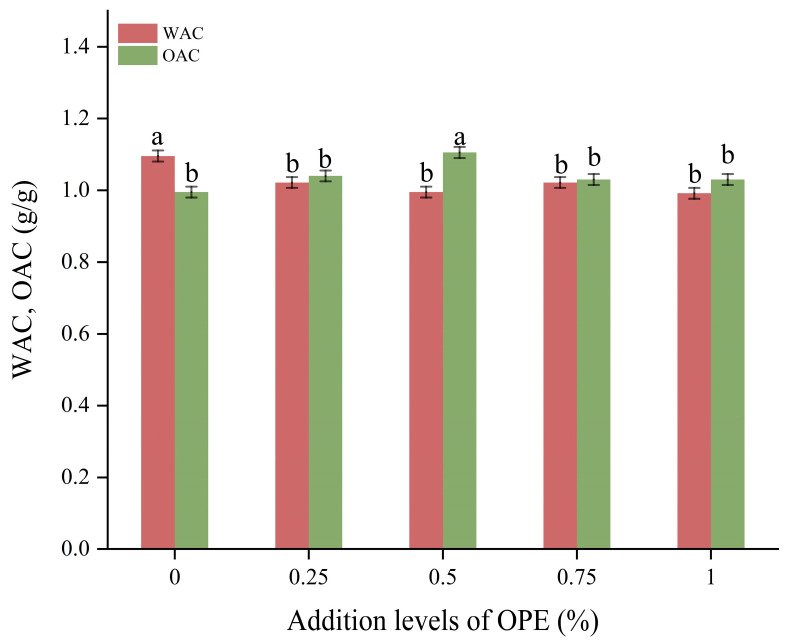
Effects of addition levels of OPE on WAC and OAC of high-gluten wheat flour. Note: Different lowercase letters indicate significant differences in values (*p* < 0.05).

Water absorption capacity (WAC) is the maximum amount of water flour can absorb without creating runoff or excess liquid. This characteristic plays a key role in determining how much water is needed for dough formation. It also affects the flavor, volume and crumb texture of the final bread product. Oil absorption capacity (OAC) is the maximum amount of oil that can be absorbed per unit mass of high-gluten wheat flour. The flakiness, softness and consistency of baked goods are all affected by their OAC. It can also shorten shelf life by affecting the rate at which the product deteriorates [23]. Figure 1. illustrates how the addition level of OPE affects the WAC and OAC of high-gluten wheat flour.

The addition of OPE led to a decrease in the WAC of high-gluten wheat flour. When the OPE addition level was 1%, the WAC of high-gluten wheat flour was the lowest, decreasing by 29.3% compared to the control group (*p* < 0.05). The reduced WAC value of high-gluten wheat flour is due to the high number of hydroxyl groups contained in the polyphenols in OPE. These groups preferentially attach to the hydrophilic protein and starch groups in high-gluten wheat flour, thus reducing the water absorption capacity of high-gluten wheat flour [33,34]. In relation to OAC, a clear pattern was noted: it first rose then fell as the concentration of OPE increased. The OAC of high-gluten wheat flour increased by 11.1% (*p* < 0.05) at 0.5% OPE, which was significantly greater than that of the control group. This may be due to the fact that starch granules have many pores extending from the surface to the center of the granule compared to untreated starch, which improves the starch’s ability to adsorb oils [35]. In addition, OPE may alter the hydrophobic interactions between oil molecules and nonpolar regions of proteins, changing the internal and external structure of wheat flour and affecting its OAC [36]. The addition level of OPE did not appreciably alter the high-gluten wheat flour’s WAC or OAC. This may be because the addition level of OPE in this study was too low to cause significant changes. Future research should focus on a wider range of OPE addition levels or use different techniques to observe more complex effects on high-gluten wheat flour properties in order to better document these changes. Similar trends were observed by Ahmad et al. [33], who studied the effect of green tea powder on the WAC and OAC of wheat flour.

### 3.3. Effect of OPE on the Falling Number of High-Gluten Wheat Flour

**Figure 2:**
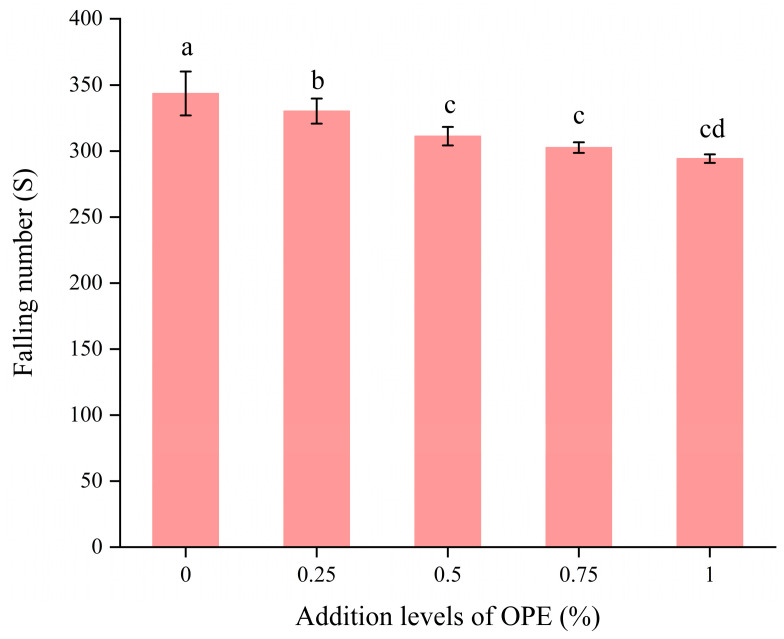
Effects of addition levels of OPE on the falling number of high-gluten wheat flour. Note: Different lowercase letters indicate significant differences in values (*p* < 0.05).

The falling number (FN), commonly used to assess flour freshness and baking quality, is a critical indicator for determining the activity of α-amylase in flour [37]. In this test, a standardized plunger is released from a predetermined height and allowed to pass through the flour paste. The FN is defined as the time (usually measured in seconds) required for the plunger to descend a specified distance. As α-amylase activity increases, the FN decreases because the enzyme breaks down starch, reducing paste viscosity and accelerating the plunger’s descent. Flour with a high FN (typically greater than 300 s), indicating low α-amylase activity and fresh flour, is suitable for producing bread and other baked goods requiring high-gluten wheat flour. Generally, high-gluten wheat flour used for bread-making should have an FN greater than 220 s [38]. Figure 2 illustrates the effect of OPE addition levels on the falling number of high-gluten wheat flour.

As the OPE addition level increased, the FN of high-gluten wheat flour gradually decreased compared to the control group. When the OPE addition level reached 14.4%, the FN decreased by 0.05% compared to the control group (*p* < 0.05). Despite this reduction, the FN of high-gluten wheat flour remained above 220 s, indicating that high-gluten wheat flour with a certain addition level of OPE still maintains suitability for bread-making. The polyphenols in OPE contain multiple hydroxyl groups that directly bind to the active sites of α-amylase, forming hydrogen bonds with key amino acid residues required for substrate recognition. This interaction sterically hinders the binding of starch to the enzyme, disrupts the catalytic mechanism, and leads to reduced α-amylase activity [39]. This inhibition of α-amylase activity slows starch decomposition, thereby decreasing the viscosity of the high-gluten wheat flour paste and causing a lower FN [40]. Therefore, although enzyme activity is reduced, high-gluten wheat flour still retains sufficient quality for bread-making [28].

### 3.4. Effect of OPE on Farinaceous Characteristics of High-Gluten Wheat Flour

Table 1 shows the effect of OPE addition levels on the farinographic properties of high-gluten wheat flour. The WA of high-gluten wheat flour decreased as the OPE addition level increased, reaching a minimum value of 59.7% when the OPE addition level was 1%. Both the development time (DT) and stability time (ST) of high-gluten wheat flour increased with higher OPE addition levels. At an OPE addition level of 1%, DT and ST showed the longest increase, rising by 286.7% and 166.1% compared to the control group, respectively. The DS of high-gluten wheat flour decreased as the OPE addition level increased, reaching the lowest DS value at 1% OPE addition level. The FI of high-gluten wheat flour increased significantly with OPE addition level (*p* < 0.05), achieving the highest FI value in the dough when the OPE addition level was 1%.

Polyphenols in OPE, especially those containing multiple hydroxyl groups, interact with hydrophilic sites (hydroxyl, amino and carboxyl groups) on high-gluten wheat flour proteins (e.g., gluten, gliadin) and starch molecules. These interactions result in the formation of strong hydrogen bonds or lead to protein aggregation, which effectively masks or occupies polar groups that could otherwise bind water. As a result, the number of hydrophilic sites available for hydration is reduced, leading to the observed decrease in WA value. The ideal dough WA range for bread baking is 51.4% to 64.2% [41]. The flour’s WA in this study was between 59.7% and 60.5%, which is within the permissible range for making bread. The addition levels of OPE had nearly no adverse effects on the dough WA of high-gluten wheat flour, nor did they significantly affect its baking suitability. Additionally, OPE incorporation increased DT, ST, and FI of high-gluten wheat flour while reducing DS of the dough. These changes indicate that OPE enhances gluten strength in the dough, thereby improving its mixing durability. Aghamirzaei et al. [42] reported that replacing part of wheat flour with grape seed powder reduced the WA of the flour. Li et al. [13] found that adding tea polyphenols decreased the WA and DS of wheat flour while increasing DT and ST. These findings are consistent with our study.

### 3.5. Effect of OPE on the Gelatinization Characteristics of High-Gluten Wheat Flour

**Figure 3:**
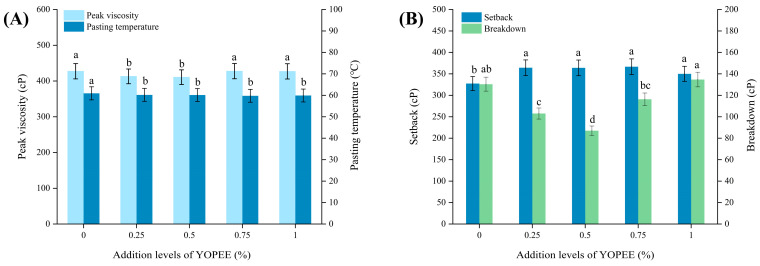
Effects of addition levels of OPE on the gelatinization characteristics of high-gluten wheat flour. Note: (**A**) illustrates Peak viscosity and Pasting temperature of high-gluten wheat flour; (**B**) illustrates Setback and Breakdown of high-gluten wheat flour. Different lowercase letters indicate significant differences in values (*p* < 0.05).

By adjusting the heating, holding, and cooling procedures of the rapid viscosity analyzer, the gelatinization characteristics of high-gluten wheat flour were investigated. The key parameters of flour gelatinization characteristics include peak viscosity, pasting temperature, setback value, and breakdown value. As shown in Figure 3A, when the OPE addition levels were 0.25% and 0.5%, the peak viscosity of high-gluten wheat flour was significantly lower than that of the control group (*p* < 0.05). At an OPE addition level of 1%, the peak viscosity of high-gluten wheat flour increased but showed no significant difference from the control group (*p* > 0.05). In contrast with the control group, OPE addition led to a significant decrease in the pasting temperature of high-gluten wheat flour (*p* < 0.05). As illustrated in Figure 3B, the setback value of the control group was 327 cP, whereas OPE addition significantly increased the setback value of high-gluten wheat flour to 364 cP. Compared with the control group, the breakdown value of high-gluten wheat flour first increased and then decreased with increasing OPE addition levels. The sample exhibited the lowest breakdown value when the OPE addition level was 0.5%.

Due to the hydrogen bonding interactions between OPE and starch molecules, as well as particle fragmentation caused by cross-linking, the peak viscosity of the samples decreased [43]. Additionally, the hydrophilic components in OPE reduce the water available for starch swelling, thereby further decreasing peak viscosity. The phenolic hydroxyl groups in the polyphenol structure can bind to starch chains via hydrogen bonds, weakening the interactions between starch molecules and thereby decreasing the pasting temperature of wheat flour [44]. OPE could also reduce the degree of starch crystallinity, thereby making the samples more prone to gelatinization [45]. OPE alters the ratio of amylose to amylopectin in wheat flour. A high proportion of amylose promotes the development of rigid, organized structures, thereby increasing the setback value of the samples [46]. At low OPE addition levels, the compound may regulate interactions between starch molecules, inhibiting excessive water absorption and swelling of starch granules during gelatinization. This results in the formation of a tighter starch paste network, leading to a lower breakdown value. In contrast, excessive OPE addition can cause over-crosslinking of starch molecules, which hinders uniform water absorption by starch granules and creates local structural defects. These effects ultimately reduce paste stability and lead to an increase in the breakdown value [47,48]. Ning et al. [49] found that the addition of green tea powder significantly reduced the peak viscosity of flour compared to the control group, which is consistent with the results of this study.

### 3.6. Effects of OPE on the Tensile Properties of High-Gluten Wheat Flour Dough

**Figure 4:**
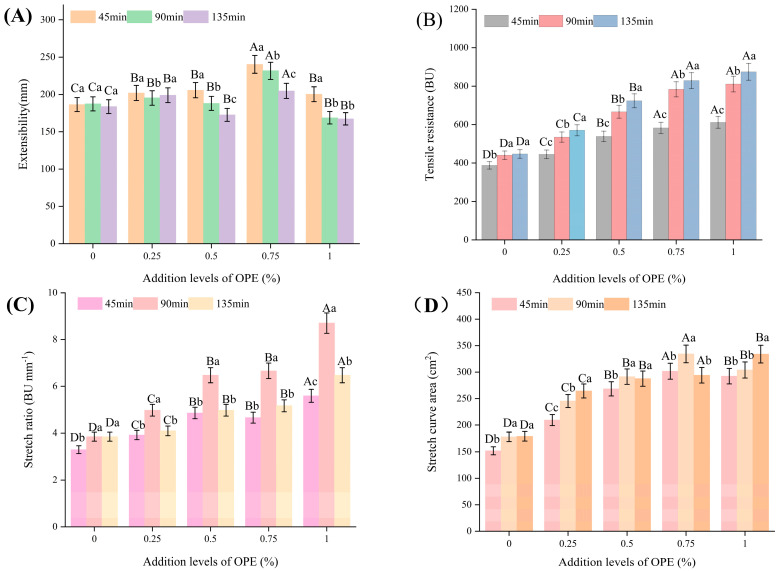
Effects of addition levels of OPE on the tensile properties of high-gluten wheat flour. Note: (**A**) illustrates Extensibility of high-gluten wheat flour dough; (**B**) illustrates Tensile resistance of high-gluten wheat flour dough; (**C**) illustrates Stretch ratio of high-gluten wheat flour dough; (**D**) illustrates Stretch curve area of high-gluten wheat flour dough. Different lowercase letters indicate significant differences in values (*p* < 0.05), and different uppercase letters indicate significant differences between the number groups (*p* < 0.05).

The tensile properties primarily reflect changes in the rheological characteristics of dough during the resting process. These properties can predict the dough’s performance during fermentation and baking, serving as an important basis for evaluating dough quality. The extensibility, tensile resistance, stretch ratio, and stretch curve area are the key parameters of tensile properties. Figure 4A–D illustrate how the addition level of OPE affects the tensile properties of high-gluten wheat flour.

As shown in Figure 4A,D, the dough extensibility and stretch curve area first increased and then decreased with the increasing OPE addition level. However, their values remained higher overall than those of the control group. As shown in Figure 4B,C, after the dough was rested for 45, 90, and 135 min, both its tensile resistance and stretch ratio significantly increased with the increasing OPE addition level. Notably, at an OPE addition level of 0.75% and a fermentation time of 45 min, the dough exhibited the best extensibility properties. The dough’s extensibility, tensile resistance, stretch curve area, and stretch ratio all increased, indicating that the addition of OPE enhanced the dough’s stability and resilience and made it easier for it to expand. The interaction between OPE’s polyphenols and gluten proteins may be the cause of this effect, as it probably fortifies the gluten network and improves the texture of the dough. It is well known that polyphenols, especially tannic acid (which is frequently referred to as a polyphenol), interact with gluten proteins through chemical bonding to form a more stable complex, which encourages the gluten network to change from “loose and disordered” to “dense and ordered”, thus enhancing the elasticity and strength of the dough at the same time [26]. Relevant research has shown that dough extensibility, tensile resistance, and stretch curve area are all improved by an increase in tannic acid content [26]. Similarly, the dough’s stretch curve area and stretch resistance showed a rising trend within the range of 0.3 to 0.9% tea polyphenol content [13].

### 3.7. Effect of OPE on Dynamic Rheological Properties of High-Gluten Wheat Flour Dough

**Figure 5:**
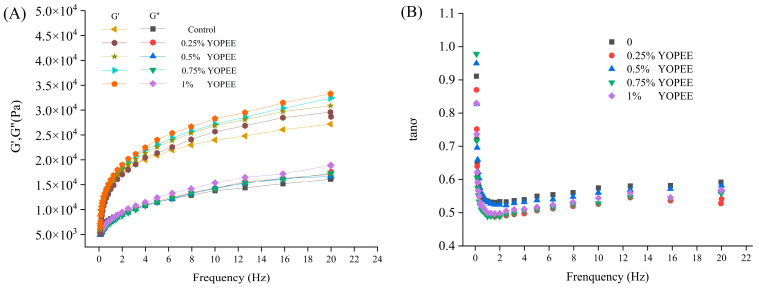
Effects of addition levels of OPE on the dynamic rheological properties of high-gluten wheat flour dough. Note: (**A**) shows elastic modulus (G′) and viscous modulus (G″); (**B**) shows the loss factor (tanσ).

The dynamic rheological properties of dough include elastic modulus (G′), viscous modulus (G″), and loss factor (tanσ). Figure 5 shows how OPE addition levels affect the dynamic rheological properties of high-gluten wheat flour dough.

As shown in Figure 5A, the G′ and G″ values of all samples increased with the increase in scanning frequency. At low scanning frequencies (≤1.0 Hz), the values of G″ and G′ were close. However, as the scanning frequency increased, G″ increased more slowly. Moreover, the gap between the values of G′ and G″ became increasingly large. Throughout the frequency sweep, G′ was always greater than G″, and tanσ (the ratio of G″ to G′) was less than 1, which indicated that the dough exhibited both solid-like elastic characteristics and liquid-like viscous characteristics, presenting an elastic gel state [21]. The G′ of the samples increased with the increasing OPE addition level, while G″ showed no significant difference with OPE addition (*p* > 0.05). The control group had the smallest G′ and G″ values. When the OPE addition level was 1%, the sample had the highest G′ and G″, suggesting that the maximum OPE addition level promoted the viscoelasticity of the dough. As shown in Figure 5B, the OPE addition levels had no significant effect on the dough’s tanσ compared to the control dough (*p* > 0.05).

### 3.8. The Total Phenolic Content, Total Flavonoid Contents, and Antioxidant Activity of Crumbs

**Figure 6:**
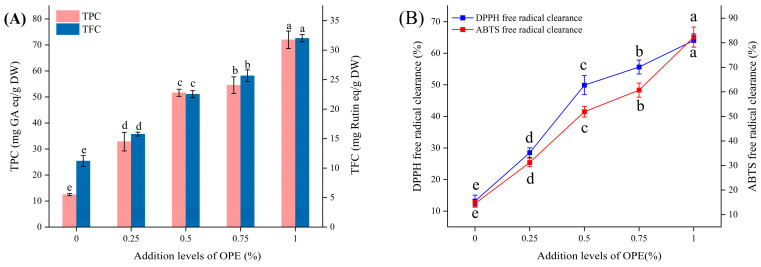
Effects of addition levels of OPE on the total phenolic content, total flavonoid contents, and antioxidant activity of crumbs. Note: (**A**) illustrates TPC and TFC of crumbs; (**B**) illustrates DPPH free radical clearance and ABTS free radical clearance of crumbs. Different lowercase letters indicate significant differences in values (*p* < 0.05).

The addition of plant polyphenols not only enhances the nutritional value of bread but also confers health benefits, thereby endowing bread with health-promoting functions. Additionally, the incorporation of polyphenolic substances in bread can improve its antioxidant properties, delay lipid oxidation, and extend the shelf life of bread [12]. The effects of OPE addition levels on the total phenolic content (TPC), total flavonoid content (TFC), and antioxidant activity of the crumbs are shown in Figure 6 and the table.

As shown in Figure 6A, when the OPE addition level was 1%, the TPC and TFC of crumbs reached their maximum values, which were 4.8 times and 1.6 times higher than those of the control group, respectively. As depicted in Figure 6B, the addition of OPE increased the antioxidant activity of crumbs. When the OPE addition level was 1%, both the DPPH and ABTS radical scavenging rates were three times those of the control group (*p* < 0.05). Sagar and Pareek [ [28] used onion peel powder when making pizza crusts. The TPC and TFC of the pizza crusts increased by 10 times and 50 times, respectively, compared to the control group (with an addition amount of 5%), while the DPPH and ABTS radical scavenging capacities increased by 50 times and 8 times, respectively.

### 3.9. Effects of OPE on the Color of Bread

Customers’ perceptions of bread quality and their purchasing decisions are directly influenced by the color of the product [50]. In this investigation, the L*, a*, and b* values of the bread were measured using a colorimeter. These values offer objective assistance for the assessment of the bread’s color by providing quantitative information on their brightness, red-green hue, and yellow-blue hue. The influence of OPE addition amount on the color of crumbs and crusts is shown in Table 2. The crumbs’ L* value decreased as the amount of OPE added increased, suggesting a darkening and mild grayish color. On the other hand, the inclusion of OPE raised the crumbs’ a* and b* values. The crumbs with 1.0% OPE (a* = 14.7, b* = 22.9) and the control group (a* = 1.3, b* = 16.0) showed the biggest variations in a* and b* values. Furthermore, there was a significant difference (*p* < 0.05) in the total color difference (ΔE) between the OPE-enriched crumbs and the control group. The crusts showed a decrease in lightness from 37.9 at 0% to 31.4 at 1%, indicating a darker crust. Meanwhile, the a* values and ΔE value of crusts increased significantly with the increase in OPE additions. This could be attributed to increased Maillard reactions in the dough matrix, which is enhanced by the presence of OPE [51]. Rodríguez et al. [52] in their study of grape pomace peels and amaranth flours on the color of bread found that the L* values of crumbs and crusts significantly decreased with the addition of grape pomace peels and amaranth flours significantly decreased with the addition of grape pomace peels and amaranth flours, while the a* values of bread crusts showed an increasing trend, which is consistent with the findings of this paper.

### 3.10. Effects of OPE on the Specific Volume of Bread

**Figure 7:**
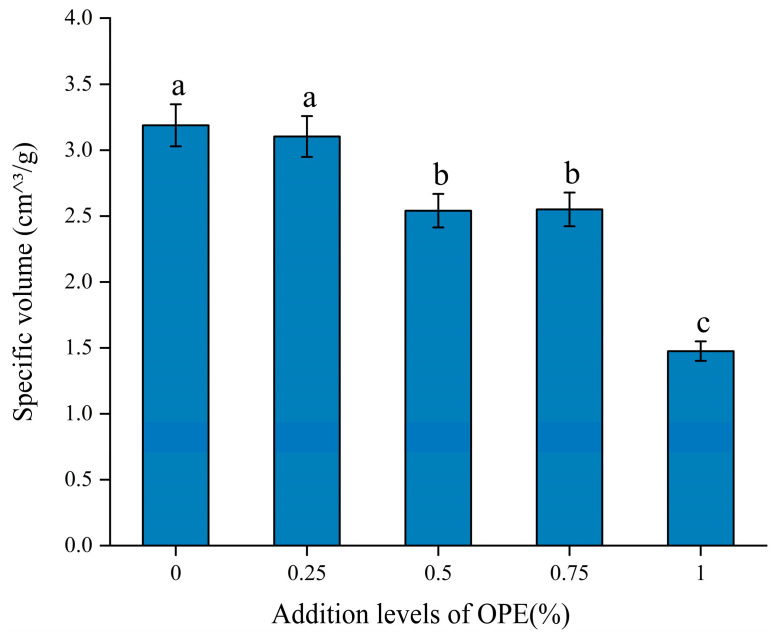
Effects of addition levels of OPE on the specific volume of bread. Note: Different lowercase letters indicate significant differences in values (*p* < 0.05).

One important measure of bread’s fluff is its specific volume, which is the volume divided by its weight [12]. In addition to assessing bread quality, this statistic offers useful information about the bread-making procedure [53]. Consumers tend to find bread with a higher specific volume more appealing, and from an economic standpoint, merchants typically favor bread with a higher specific volume. Figure 7 shows a visual comparison of bread with and without OPE.

As the OPE addition level increased, the specific volume of the bread decreased. When the OPE addition level was 1%, the specific volume of the bread decreased by 52% compared to the control group. This reduction may be due to the polyphenols in OPE weakening the formation and strength of gluten, resulting in fewer pores in the bread and an increase in the surface area of the pores, which in turn leads to a decrease in the specific volume of the bread [12]. Additionally, the polyphenols in OPE interact with the secondary structure of gluten protein, weakening the gluten network and making it more susceptible to breakage. The attenuated gas retention capacity during fermentation induced compromised CO₂ entrapment capability, which directly precipitated a reduction in bread specific volume [54]. Wang et al. [12], who incorporated tea polyphenols into wheat flour, and Chen et al. [55], who added green tea extract to flour, both reported a decrease in bread specific volume, which is in line with the results of this investigation.

### 3.11. Effects of OPE on the Textural Properties of Crumbs

The texture quality of a product typically indicates its shelf life and consumer acceptance [56]. Texture analysis of bread involves simulating the process of teeth chewing to evaluate the degree of reaction to external forces during the chewing process [57]. Texture analysis includes key parameters such as the hardness, chewiness, springiness, and cohesiveness of the sample. Hardness is related to the force required to bite the bread and is often used as an indicator to determine bread quality [58]. Cohesiveness quantifies the internal resistance and cohesion of the bread [59], while springiness reflects the degree of bread staling [60]. Chewiness refers to the difficulty or comfort of chewing the bread in the mouth, where higher values indicate greater chewing difficulty [15]. The effects of OPE addition levels on the texture properties of crumbs are shown in Table 3.

The hardness and chewiness of crumbs first decreased and then increased with the increasing OPE addition level. When the OPE addition level was 0.25~0.75%, the hardness and chewiness of crumbs were significantly lower than those of the control (*p* < 0.05). This may be because polyphenols disrupt the disulfide bonds in gluten proteins, weakening gluten strength and thus reducing bread hardness [26]. When the OPE addition level was 1%, the hardness and chewiness of t crumbs were significantly higher than those of the control (*p* < 0.05). Studies have shown a significant negative correlation between bread’s specific volume and crumbs’ hardness [61]. Peng et al. [62] investigated the effect of grape seed extract on bread hardness and found that bread hardness first decreased and then increased after extract addition, consistent with the results of this study. As the OPE addition level increased, the springiness of crumbs gradually decreased. This may be attributed to non-covalent interactions between polyphenols and gluten proteins, leading to rearrangement of the secondary structure of gluten and weakening of the gluten network, thereby reducing bread springiness [63]. Zahorec et al. [15] observed a decrease in crumbs’ springiness with increasing carob extract content when studying its effect on bread texture, which aligns with our findings. The cohesiveness of crumbs was significantly lower than that of the control as the OPE addition level increased (*p*< 0.05), reaching the lowest value at an OPE addition level of 1%. Reduced cohesiveness indicates lower internal resistance in the bread structure and weaker internal bonding strength between bread components, making the bread prone to crushing during chewing [64]. This is likely due to the disruption of the gluten network with higher OPE addition, leading to decreased gas-holding capacity and a looser internal structure, thus reducing crumb cohesiveness. Wang et al. [12] reached similar conclusions in their study on the effect of tea polyphenols on bread texture.

### 3.12. Effects of OPE on the Microstructure of Bread

**Figure 8:**
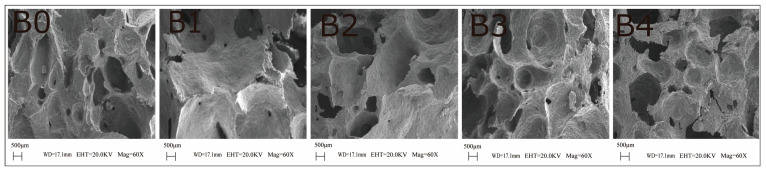
Scanning electron microscope images of bread with different addition levels of OPE. Note: (**B0**–**B4**) are breads prepared with 0%, 0.25%, 0.5%, 0.75%, and 1% replacement of high-gluten wheat flour with onion peel extract, respectively.

Scanning electron microscopy (SEM) was used to examine the microstructure of bread cross-sections to evaluate the effect of OPE on gluten network formation in the bread matrix. Figure 8 shows the acquired images.

The control group formed a loose network structure, while the structure of bread with different OPE addition levels changed to varying degrees. Compared with the control, the bread with a 0.25% OPE addition level had a more loosely structured network, with relatively continuous and intact pores observable. However, as the OPE addition level increased, the bread structure was gradually disrupted. When the OPE addition level reached 1%, the continuity and integrity of internal pores in the bread were severely affected; the pore walls became rough, and the gluten matrix was partially aggregated or interrupted by starch granules. This may be because excessive OPE may have a diluting effect on gluten proteins, resulting in an uneven gluten structure and a decrease in dough strength. Additionally, the polyphenols in OPE interact with the secondary structure of gluten protein, weakening the gluten network and making it more susceptible to breakage. The attenuated gas retention capacity during fermentation induced compromised CO₂ entrapment capability, which directly precipitated a reduction in the bread’s specific volume. Liu et al. [65] found that the addition of gallic acid to steamed bread caused deterioration of the gluten network, leading to irregular holes and cracks. Additionally, Sezin and Şek [18] studied the effect of rose flower extract on the microstructure of baked bread and observed that the control group had a more extensive gluten network, whereas the addition of rosehip extract containing phenolic acids disrupted the initially continuous gluten network. These results are consistent with the findings of the current study.

### 3.13. Effects of OPE on the Sensory Evaluation of Bread

**Figure 9:**
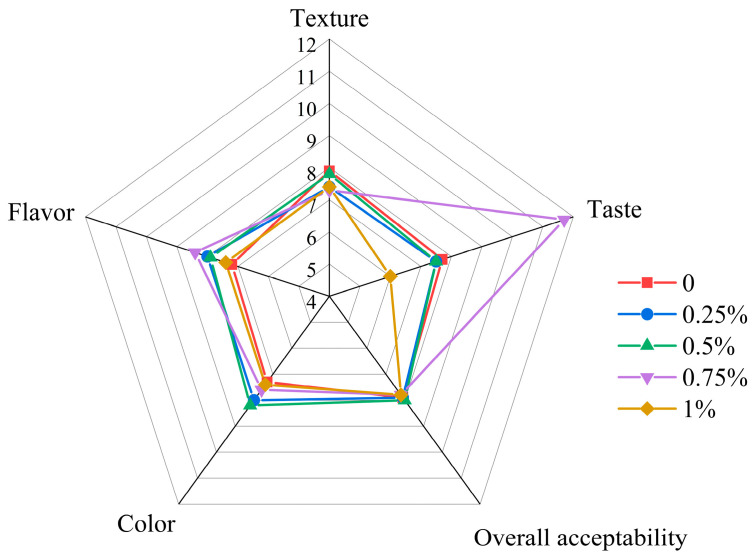
Spider web graph of sensory attributes of bread incorporated with onion peel extract at different addition levels. Note: Each value is expressed as mean ± standard deviation (*n* = 50). The value of each hedonic scale is the average of 50 repetitions. Values are on a 9-point hedonic scale, where 1, 5, and 9 represent “really dislike”, “neither like nor dislike”, and “really like” respectively.

Bread’s flavor, color, taste, texture, and overall acceptability are all sensory evaluation indicators. OPE strengthens bread, as shown by the instrumental study previously reported, but its sensory qualities must also be advantageous for customer acceptability. Customers are likely to reject the addition of OPE if it adversely affects these attributes. As a result, sensory analysis is essential for the creation of new goods [15]. Table 4 and Figure 9 display the bread’s sensory evaluation results. Compared with the control group, breads with OPE addition levels of 0.25%, 0.5%, and 0.75% showed slightly higher scores in flavor and color. It was considered that bread with appropriate OPE addition had a more appetizing color and a unique flavor, especially the bread with 0.75% OPE, which was widely favored by consumers in terms of taste. When OPE was added at moderate levels (0.25–0.75%), no significant differences were observed in bread texture and overall acceptability compared with the control group. However, bread with 1% OPE addition showed significantly lower scores in texture and taste; panelists noted higher hardness, poor chewiness, and a pronounced bitter taste, leading to the lowest overall acceptability. In the scoring system, “5” represented a neutral value (“neither like nor dislike”), with scores above 5 indicating acceptance and below 5 indicating non-acceptance. As shown in Figure 9, all samples scored above 5. The scores for bread texture and color were consistent with the measurements from physical tests, verifying the reliability of the sensory evaluation results.

## 4. Conclusions

This study systematically evaluated the effect of ethanolic extract of yellow onion peel (OPE) on the functional, rheological, and sensory properties of high-gluten wheat flour and its application in bread-making. The main findings indicated that the addition level of 0.5–0.75% OPE enhanced the integrity of the gluten network, improved dough processing stability (e.g., enhancement of mechanical resistance and starch pasting), and increased the antioxidant capacity of bread qualities, which is in line with the growing consumer demand for nutritious and functional bakery products. The unique phenolic profile of OPE also introduces desirable organoleptic attributes; for example, a moderate amount of onion skin extract adds to the bread’s aroma and attractive color while promoting a denser, more uniform crumb structure. However, the addition of OPE can impair bread elasticity and cohesion and reduce specific volume, highlighting the trade-off between health benefits and structural quality. The use of OPE as a natural functional additive offers the potential for the development of antioxidant-rich bakery products without synthetic preservatives, meeting the market demand for waste-utilized, nutritionally enhanced foods. Its ability to improve dough rheology may also support the formulation of whole grain or gluten-modified breads, where structural stability is critical. To realize the full potential of OPE, future research should be conducted by investigating its synergistic effects with emulsifiers or yeast to mitigate adverse effects on the physical properties of bread (e.g., low specific volume) while retaining the benefits of polyphenols. In conclusion, this study identifies onion skin extract as a promising natural ingredient for functional bread-making, and future research is essential to overcome technological limitations and unlock its full potential in sustainable and healthy food systems.

## Figures and Tables

**Table 1:** Effects of addition levels of OPE on the farinaceous characteristics of high-gluten wheat flour.

Addition Levels of OPE (%)	WA (%)	DT (min)	ST (min)	DS (FU)	FI
0	60.5 ± 0.3 ^a^	7.5 ± 1.1 ^b^	10.9 ± 0.1 ^e^	70.0 ± 6.1 ^a^	136.7 ± 15.0 ^d^
0.25	60.2 ± 0.2 ^b^	8.7 ± 1.1 ^b^	12.5 ± 0.5 ^d^	53.3 ± 1.5 ^b^	138.7 ± 17.6 ^d^
0.5	60.1 ± 0.2 ^bc^	10.2 ± 0.4 ^a^	15.3 ± 1.2 ^c^	49.3 ± 4.6 ^b^	172.0 ± 4.4 ^c^
0.75	59.9 ± 0.5 ^c^	10.3 ± 0.5 ^a^	21.1 ± 0.9 ^b^	35.0 ± 3.6 ^c^	213.3 ± 16.9 ^b^
1	59.7 ± 0.1 ^c^	11.4 ± 0.1 ^a^	29.0 ± 0.1 ^a^	18.0 ± 1.0 ^d^	271.0 ± 4.7 ^a^

Data are means plus standard deviation (*n* = 3). Data with different alphabets in the same column are significantly different (*p* < 0.05). Note: WA (%) = water absorption, DT (min) = development time, ST (min) = stability time, DS (FU) = degree of softening, FI = farinograph index. Different lowercase letters indicate significant differences in values (*p* < 0.05).

**Table 2:** Effects of addition levels of OPE on crumb and crust color.

Addition Levels of OPE		L*	a*	b*	ΔE	Picture
Crumbs	0	83.7 ± 0.3 ^a^	1.3 ± 0.1 ^d^	16.0 ± 0.1 ^b^	0	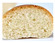
	0.25%	67.6 ± 1.4 ^b^	10.5 ± 0.3 ^c^	21.0 ± 0.2 ^a^	15.7 ± 1.2 ^a^	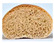
	0.5%	66.1 ± 0.3 ^c^	13.3 ± 0.8 ^b^	22.7 ± 1.5 ^a^	21.1 ± 0.8 ^b^	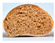
	0.75%	65.6 ± 0.3 ^c^	13.7 ± 0.2 ^b^	22.8 ± 0.6 ^a^	25.8 ± 1.7 ^b^	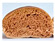
	1%	62.2 ± 0.3 ^d^	14.7 ± 0.2 ^a^	22.9 ± 0.3 ^a^	34.0 ± 2.3 ^c^	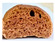
Crusts	0	37.9 ± 3.6 ^a^	9.9 ± 1.4 ^c^	19.6 ± 5.1 ^a^	/	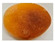
	0.25%	33.5 ± 2.0 ^b^	10.5 ± 1.5 ^c^	19.5 ± 1.9 ^a^	7.6 ± 1.2 ^c^	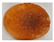
	0.5%	32.5 ± 2.7 ^b^	12.6 ± 2.4 ^b^	18.3 ± 0.7 ^a^	9.3 ± 3.1 ^b^	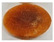
	0.75%	32.6 ± 0.7 ^b^	13.7 ± 0.5 ^a^	18.2 ± 0.8 ^a^	11.7 ± 2.1 ^a^	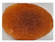
	1%	31.4 ± 1.5 ^c^	14.6 ± 2.3 ^a^	18.6 ± 4.4 ^a^	11.0 ± 2.1 ^a^	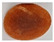

Note: Data are expressed as mean ± SD. Different lowercase letters in the same column indicate significant differences between different treatments (*p* < 0.05).

**Table 3:** Effects of addition levels of OPE on the textural properties of crumbs.

Addition Levels of OPE (%)	Hardness (N)	Chewiness (N)	Springiness (min)	Cohesiveness
0	3.8 ± 0.1 ^b^	18.4 ± 0.9 ^b^	6.8 ± 0.1 ^a^	0.8 ± 0.02 ^a^
0.25	1.7 ± 0.9 ^d^	8.2 ± 0.8 ^d^	6.3 ± 0.1 ^b^	0.7 ± 0.06 ^b^
0.5	2.7 ± 0.2 ^c^	10.3 ± 1.0 ^c^	6.0 ± 0.3 ^c^	0.6 ± 0.08 ^c^
0.75	4.2 ± 0.2 ^b^	19.9 ± 1.2 ^b^	5.9 ± 0.1 ^c^	0.6 ± 0.01 ^c^
1	5.7 ± 0.1 ^a^	28.1 ± 0.8 ^a^	5.9 ± 0.2 ^c^	0.5 ± 0.02 ^d^

Note: Data are expressed as mean ± SD. Different lowercase letters in the same column indicate significant differences between different treatments (*p* < 0.05).

**Table 4:** Effects of addition levels of OPE on the sensory attributes of crumbs.

Addition Levels of OPE (%)	Texture	Taste	Color	Flavor	Overall Acceptability
0	7.9 ± 0.1 ^a^	7.7 ± 0.1 ^b^	7.3 ± 0.3 ^a^	7.2 ± 0.1 ^a^	7.9 ± 0.1 ^a^
0.25	7.4 ± 0.4 ^a^	7.5 ± 0.2 ^b^	8.0 ± 0.1 ^a^	8.0 ± 0.1 ^a^	7.9 ± 0.9 ^a^
0.5	7.8 ± 0.2 ^a^	7.5 ± 0.3 ^b^	8.2 ± 0.2 ^a^	7.9 ± 0.2 ^a^	8.0 ± 0.2 ^a^
0.75	7.3 ± 0.8 ^a^	11.7 ± 0.4 ^a^	7.6 ± 0.3 ^a^	8.4 ± 0.4 ^a^	7.8 ± 0.6 ^a^
1	7.4 ± 0.1 ^a^	6.0 ± 0.2 ^c^	7.4 ± 0.8 ^ab^	7.4 ± 0.8 ^a^	7.8 ± 0.3 ^a^

Note: Data are expressed as mean ± SD. Different lowercase letters in the same column indicate significant differences between different treatments (*p* < 0.05).

## Data Availability

The original contributions presented in this study are included in the article. Further inquiries can be directed to the corresponding authors.

## Supplementary Materials

The following supporting information can be downloaded at: https://www.mdpi.com/article/10.3390/foods14091618/s1, The content of the supplementary materials mainly consists of the specific steps of the determination method.

## References

[1] Topolska K., Florkiewicz A., Filipiak-Florkiewicz A. (2021). Functional food—Consumer motivations and expectations. Int. J. Environ. Res. Public Health.

[2] Mickiewicz B., Britchenko I. (2022). Main trends and development forecast of bread and bakery products market. VUZF Rev..

[3] Li Y., Ma D., Sun D., Wang C., Zhang J., Xie Y., Guo T. (2015). Total phenolic, flavonoid content, and antioxidant activity of flour, noodles, and steamed bread made from different colored wheat grains by three milling methods. Crop. J..

[4] Czaja A., Czubaszek A., Wyspiańska D., Sokół-Łętowska A., Kucharska A.Z. (2020). Quality of wheat bread enriched with onion extract and polyphenols content and antioxidant activity changes during bread storage. Int. J. Food Sci. Technol..

[5] World Health Organization (2022). Guideline: Fortification of Wheat Flour with Vitamins and Minerals as a Public Health Strategy.

[6] Yu L., Nanguet A.-L., Beta T. (2013). Comparison of antioxidant properties of refined and whole wheat flour and bread. Antioxidants.

[7] Gómez M., Gutkoski L.C., Bravo-Núñez Á. (2020). Understanding whole-wheat flour and its effect in breads: A review. Compr. Rev. Food Sci. Food Saf..

[8] Lipșa F.D., Stoica F., Rațu R.N., Veleșcu I.D., Cârlescu P.M., Motrescu I., Usturoi M.G., Râpeanu G. (2024). Red onion peel powder as a functional ingredient for manufacturing ricotta cheese. Foods.

[9] Majeed S., Ali M., Malik A.M. (2024). Optimizing Efficiency: Mitigating Food Loss and Waste in Vegetable Supply Chain. Pak. Res. J. Soc. Sci..

[10] Jaganmohanrao L. (2025). Valorization of onion wastes and by-products using deep eutectic solvents as alternate green technology solvents for isolation of bioactive phytochemicals. Food Res. Int..

[11] Munir M., Kheirkhah H., Baroutian S., Quek S.Y., Young B.R. (2018). Subcritical water extraction of bioactive compounds from waste onion skin. J. Clean. Prod..

[12] Qin W., Pi J., Zhang G. (2022). The interaction between tea polyphenols and wheat gluten in dough formation and bread making. Food Funct..

[13] Li H., Ma J., Zhao B., Pan L., Meng J., Xu B. (2020). Effect of tea polyphenols on the quality characteristics of fresh wheat noodles in the storage. Int. J. Food Sci. Technol..

[14] Xiao Z., Li R., Luo Z., Duan Y., Zhang H., Liu L., Lü C., Yang Q. (2021). Effect of modified wheat bran on the structure and digestibility of bread. Food Chem..

[15] Zahorec J., Šoronja-Simović D., Petrović J., Šereš Z., Pavlić B., Sterniša M., Smole Možina S., Ačkar Đ., Šubarić D., Jozinović A. (2024). The Effect of Carob Extract on Antioxidant, Antimicrobial and Sensory Properties of Bread. Appl. Sci..

[16] Bao Z., Fan M., Hannachi K., Li T., Zhao J., Li Y., Qian H., Wang L. (2023). Antifungal activity of star anise extract against Penicillium roqueforti and Aspergillus niger for bread shelf life. Food Res. Int..

[17] Gumul D., Ziobro R., Korus J., Kruczek M. (2021). Apple pomace as a source of bioactive polyphenol compounds in gluten-free breads. Antioxidants.

[18] Şimşek S.T., Özgen S. (2023). Vacuum modification of wheat bread with encapsulated rosehip extract: Evaluation of physicochemical and microstructural properties. J. Cereal Sci..

[19] Kan L., Oliviero T., Verkerk R., Fogliano V., Capuano E. (2020). Interaction of bread and berry polyphenols affects starch digestibility and polyphenols bio-accessibility. J. Funct. Foods.

[20] Al-Marazeeq K.M., Al-Rousan W., Al-obaidy K., Al-obaidy M. (2019). The effect of using water sumac (*Rhus coriaria* L.) extract on wheat pan bread quality characteristics. Cereal Chem..

[21] Zhang M., Peng H., Li B., Tian J. (2023). Impact of pomegranate fruit powder on dough, textural and functional properties of fresh noodle. J. Sci. Food Agric..

[22] da Cruz E.P., Jansen E.T., de Vasconcelos Costa L., de Souza E.J.D., Fonseca L.M., Gandra E.A., da Rosa Zavareze E., Dias A.R.G. (2023). Use of red onion skin (*Allium cepa* L.) in the production of bioactive extract and application in water-absorbing cryogels based on corn starch. Food Hydrocoll..

[23] Okwunodulu I., Egbuta G., Okwunodulu F., Ojinnaka C., Onyeiwu S. (2024). Gluten free sourdough breads from pearl millet-Bambara nut and pearl millet-soybean paste: Evaluation of the proximate, functional properties of the flour blends and the bread nutritional indexes. Food Chem. Adv..

[24] Graybosch R., Gang Guo G.G., Shelton D. (2000). Aberrant falling numbers of waxy wheats independent of α-amylase activity. Cereal Chem..

[25] Wu Y., Niu M., Xu H. (2020). Pasting behaviors, gel rheological properties, and freeze-thaw stability of rice flour and starch modified by green tea polyphenols. LWT.

[26] Zhang L., Cheng L., Jiang L., Wang Y., Yang G., He G. (2010). Effects of tannic acid on gluten protein structure, dough properties and bread quality of Chinese wheat. J. Sci. Food Agric..

[27] Bedrníček J., Jirotková D., Kadlec J., Laknerová I., Vrchotová N., Tříska J., Samková E., Smetana P. (2020). Thermal stability and bioavailability of bioactive compounds after baking of bread enriched with different onion by-products. Food Chem..

[28] Sagar N.A., Pareek S. (2020). Dough rheology, antioxidants, textural, physicochemical characteristics, and sensory quality of pizza base enriched with onion (*Allium cepa* L.) skin powder. Sci. Rep..

[29] Tang S., Su T., Huang Z., Huang F., Zhang R., Dong L., Deng M., Shen Y., Su D. (2022). Impact of replacing wheat flour with lychee juice by-products on bread quality characteristics and microstructure. LWT.

[30] Singh V., Krishan P., Shri R. (2017). Extraction of antioxidant phytoconstituents from onion waste. J. Pharmacogn. Phytochem..

[31] Pobłocka-Olech L., Głód D., Żebrowska M.E., Sznitowska M., Krauze-Baranowska M. (2016). TLC determination of flavonoids from different cultivars of Allium cepa and Allium ascalonicum. Acta Pharm..

[32] Lewoyehu M., Amare M. (2019). Comparative evaluation of analytical methods for determining the antioxidant activities of honey: A review. Cogent Food Agric..

[33] Ahmad M., Baba W.N., Wani T.A., Gani A., Gani A., Shah U., Wani S., Masoodi F. (2015). Effect of green tea powder on thermal, rheological & functional properties of wheat flour and physical, nutraceutical & sensory analysis of cookies. J. Food Sci. Technol..

[34] Kaur M., Singh V., Kaur R. (2017). Effect of partial replacement of wheat flour with varying levels of flaxseed flour on physicochemical, antioxidant and sensory characteristics of cookies. Bioact. Carbohydr. Diet. Fibre.

[35] Liu Y., Li M., Zhu C., Wei M. (2023). Effect of synergic pretreatment with ultrasound and alkaline hydrogen peroxide on enzymolysis and physicochemical properties of corn starch. Biomass-Convers. Biorefinery.

[36] Dhillon B., Choudhary G., Sodhi N.S. (2020). A study on physicochemical, antioxidant and microbial properties of germinated wheat flour and its utilization in breads. J. Food Sci. Technol..

[37] Jukić M., Šumanovac F., Nakov G., Šimić G., Komlenić D.K., Ivanova N., Lukinac J. (2023). Application of the falling number method in the evaluation of the α-amylase activity of malt flour. Appl. Sci..

[38] Hui Y.H., Corke H., De Leyn I., Nip W.-K., Cross N.A. (2014). Bakery Products: Science and Technology.

[39] Sun L., Warren F.J., Gidley M.J. (2019). Natural products for glycaemic control: Polyphenols as inhibitors of alpha-amylase. Trends Food Sci. Technol..

[40] Coţovanu I., Mironeasa S. (2022). Features of bread made from different amaranth flour fractions partially substituting wheat flour. Appl. Sci..

[41] Wang F., Sun X. (2002). Creep-recovery of wheat flour doughs and relationship to other physical dough tests and breadmaking performance. Cereal Chem..

[42] Aghamirzaei M., Peighambardoust S., Azadmard-Damirchi S., Majzoobi M. (2015). Effects of grape seed powder as a functional ingredient on flour physicochemical characteristics and dough rheological properties. J. Agric. Sci. Technol..

[43] Tacer-Caba Z., Nilufer-Erdil D., Boyacioglu M., Ng P. (2016). Effect of wheat protein isolate addition on the quality of grape powder added wheat flour extrudates. Qual. Assur. Saf. Crop. Foods.

[44] Du J., Yang Z., Xu X., Wang X., Du X. (2019). Effects of tea polyphenols on the structural and physicochemical properties of high-hydrostatic-pressure-gelatinized rice starch. Food Hydrocoll..

[45] Chen Y., Chen M., Wang H., Zhao S. (2014). Molecular weight distribution, structures and microscopic morphology of several different starches. J. Chin. Cereals Oils Assoc..

[46] Biduski B., da Silva W.M.F., Colussi R., El Halal S.L.d.M., Lim L.-T., Dias Á.R.G., da Rosa Zavareze E. (2018). Starch hydrogels: The influence of the amylose content and gelatinization method. Int. J. Biol. Macromol..

[47] She Z., Zhao Q., Hou D., Wang J., Lan T., Sun X., Ma T. (2024). Partial substitution of wheat flour with kiwi starch: Rheology, microstructure changes in dough and the quality properties of bread. Food Chem. X.

[48] Canalis M.B., Leon A.E., Ribotta P.D. (2019). Incorporation of dietary fiber on the cookie dough. Effects on thermal properties and water availability. Food Chem..

[49] Ning J., Hou G.G., Sun J., Wan X., Dubat A. (2017). Effect of green tea powder on the quality attributes and antioxidant activity of whole-wheat flour pan bread. LWT.

[50] Hussein A.S., Mostafa S., Fouad S., Hegazy N.A., Zaky A.A. (2023). Production and evaluation of gluten-free pasta and pan bread from Spirulina Algae powder and quinoa flour. Processes.

[51] Indiarto R., Reni R., Utama G.L., Subroto E., Pangawikan A.D., Djali M. (2023). The physicochemical, antioxidant, and sensory properties of chocolate biscuits incorporated with encapsulated mangosteen (*Garcinia mangostana* L.) peel extract. Int. J. Food Prop..

[52] Rodríguez M., Bianchi F., Simonato B., Rizzi C., Fontana A., Tironi V.A. (2024). Exploration of grape pomace peels and amaranth flours as functional ingredients in the elaboration of breads: Phenolic composition, bioaccessibility, and antioxidant activity. Food Funct..

[53] Verdonck C., De Bondt Y., Pradal I., Bautil A., Langenaeken N.A., Brijs K., Goos P., De Vuyst L., Courtin C.M. (2023). Impact of process parameters on the specific volume of wholemeal wheat bread made using sourdough-and baker’s yeast-based leavening strategies. Int. J. Food Microbiol..

[54] Verheyen C., Albrecht A., Elgeti D., Jekle M., Becker T. (2015). Impact of gas formation kinetics on dough development and bread quality. Food Res. Int..

[55] Chen G., Wang J., Li Y. (2022). Extracts of sorghum bran, grape seed, and green tea: Chromatographic comparison of phenolic profiles and mitigation effect on acrylamide in antioxidant-fortified bread. Food Chem. Adv..

[56] Homyuen A., Vanitjinda G., Yingkamhaeng N., Sukyai P. (2023). Microcrystalline Cellulose Isolation and Impregnation with Sappan Wood Extracts as Antioxidant Dietary Fiber for Bread Preparation. ACS Omega.

[57] Ma Q., Cai S., Jia Y., Sun X., Yi J., Du J. (2020). Effects of hot-water extract from vine tea (*Ampelopsis grossedentata*) on acrylamide formation, quality and consumer acceptability of bread. Foods.

[58] Bourne M. (2002). Food Texture and Viscosity: Concept and Measurement.

[59] Jiang G., Feng X., Wu Z., Li S., Bai X., Zhao C., Ameer K. (2021). Development of wheat bread added with insoluble dietary fiber from ginseng residue and effects on physiochemical properties, in vitro adsorption capacities and starch digestibility. LWT.

[60] Matos M.E., Rosell C.M. (2012). Relationship between instrumental parameters and sensory characteristics in gluten-free breads. Eur. Food Res. Technol..

[61] Cacak-Pietrzak G., Dziki D., Gawlik-Dziki U., Sułek A., Wójcik M., Krajewska A. (2022). Dandelion flowers as an additive to wheat bread: Physical properties of dough and bread quality. Appl. Sci..

[62] Peng X., Ma J., Cheng K.-W., Jiang Y., Chen F., Wang M. (2010). The effects of grape seed extract fortification on the antioxidant activity and quality attributes of bread. Food Chem..

[63] Pan J., Zhang H., Liu J., Jiang Y., Lv Y., Han J. (2021). Effects of catechins on the polymerisation behaviour, conformation and viscoelasticity of wheat gluten. Int. J. Food Sci. Technol..

[64] Ni Q., Ranawana V., Hayes H.E., Hayward N.J., Stead D., Raikos V. (2020). Addition of broad bean hull to wheat flour for the development of high-fiber bread: Effects on physical and nutritional properties. Foods.

[65] Liu S., Sun H., Ma M., Mu T. (2024). Effects of dietary fibres, polyphenols and proteins on the estimated glycaemic index, physicochemical, textural and microstructural properties of steamed bread. Int. J. Food Sci. Technol..

