# Supplementary material for: Influence of Onion Peel Extract on the Dough Characteristics of High-Gluten Wheat Flour and the Quality of Bread
Source: Foods. 2025 May 3;14(9):1618. doi: 10.3390/foods14091618 (PMC12071264; doi:10.3390/foods14091618)
Supplement: Supplementary file 1 [file foods-14-01618-s001.zip › foods-3581619-supplementary.pdf]

# Supplementary File

**S2. Materials and Methods**

High-gluten wheat flour (without any additives) was purchased from the local supermarket. The yellow onion peel was provided by the farmers' market in Qiqihar, China (without any signs of mold infection). Prior to extraction, the yellow onion peel was thoroughly washed to remove surface dirt, debris, and other contaminants, then dried in an electric blast drying oven at 40 °C. After drying, the material was ground using a crusher and passed through a 100-mesh sieve for further use. Sodium nitrite, sodium hydroxide, sodium carbonate, aluminum chloride, quercetin, Folin-Ciocalteu reagent, gallic acid, DPPH (2,2-diphenyl-1-picrylhydrazyl),potassiumpersulfate,ABTS(2,2′-azino-bis-3-ethylbenzothiazoline-6-sulphonic acid), and other chemicals were purchased from Shanghai Eon Chemical Technology Co., Ltd., China.

*S2.1. Preparation of the Onion Peel Extract*

The preparation of onion peel extract (OPE) was referred to the method of da et al. [22] with slight modifications. The entire process consisted of two extraction cycles per sample (first extraction of 50 g of powder with 500 mL of 70% ethanol, followed by filtration, centrifugation and collection of the supernatant). After mixing the supernatants from two cycles of each experiment, the extracts were concentrated by vacuum evaporation at 50°C to remove ethanol, and the resulting aqueous concentrate was lyophilized (freeze-dried) without additional solvents to obtain the final powder. The procedure was repeated until enough OPE was obtained to conduct the experiments.

*S2.2. Quality Determination of High-Gluten Wheat Flour High-Gluten Wheat Flour with Different Levels of OPE*

S2.2.1. Determination of Water Absorption Capacity and Oil Absorption Capacity

The water absorption capacity and oil absorption capacity of high-gluten wheat flour with different levels of OPE were evaluated following the procedures outlined by Okwunodulu et al [22]. 2 g of high-gluten wheat flour was dispersed in 40 ml of distilled water or refined oil and the samples were vortexed for 10 min. Afterwards, the samples were centrifuged at 4000 g for 20 min (25 °C). After removing the supernatant (distilled water or refined oil), the centrifuge tubes were inverted on paper towels to drain for 10 min and the residue was weighed. WAC and OAC were calculated and expressed as grams of water or oil absorbed per gram of sample, respectively. The test results were averaged over three determinations.

S2.2.2. Determination of Falling Number

The falling number of high-gluten wheat flour with different levels of OPE was measured using a falling number tester (Hangzhou Daji Photoelectric Instrument Co., Ltd., China), as described by Graybosch et al [23].

S2.2.3. Determination of Farinograph Characteristics

Farinograph characteristics of high-gluten wheat flour with different levels of OPE were determined according to Zhang et al [20]. using the farinograph (Beijing Dongfu Jiuheng Instrument Technology Co., Ltd., China). Weigh 300 g of mixed flour (add 0%, 0.25%, 0.5%, 0.75%, 1% of the flour weight of OPE, respectively) into the mixing tank of the powder quality instrument. The program reaction temperature 30 °C, pre-mixing time 1 min, water addition 61%, stirring speed 60 r/min. After starting the program to start pre-mixing, pre-mixing end automatically add 12.2 mL of water. After the addition of water, the plastic

plate was covered to prevent the water from evaporating, and the stirring was stopped for 18 min. The analysis included water absorption, development time, stability time, degree of soften-ing, and farinograph number, as supported by the instrument's software.

S2.2.4. Determination of Gelatinization Characteristics

The gelatinization properties of high-gluten wheat flour with different levels of OPE were determined using a rapid viscosity analyzer (Tianjin Fulutong Technology and Trade Co., Ltd., China) ac-cording to the method of Wu et al [24]. Pasting temperature, peak viscosity, breakdown, and setback were analyzed via the instrument's software.

S2.2.5. Determination of Tensile Properties

Tensile properties of high-gluten wheat flour with different levels of OPE were assessed following the procedure described by Zhang et al. [25], using an extensograph (Beijing Dongfu Jiuheng Instrument Technology Co., Ltd., China). Take 5 portions of 30 g each of high-gluten wheat flour with different contents of OPE (0%, 0.25%, 0.5%, 0.75%, 1%, g/g), and mix with appropriate amount of water for 5 min to obtain the dough. Before being put in the dough preparation tank of the mass spectrometer's tensile testing probe, the dough was allowed to rise at 30 °C for 45, 90, and 135 minutes, respectively. The dough strips of 2 mm (width) x 60 mm (length) were pressed out using the accessory equipment of the probe (non-sticky polytetrafluoroethylene plate). To ascertain the strips' tensile characteristics, they were promptly taken out of the dough preparation tank and put on the installed tensile probe. The resistance to stretching - distance to stretching curve of the dough was obtained from the start of the test until the dough broke. The parameters of the study were the maximum stretching resistance, the stretching distance, and the area of the stretching curve.

S2.2.6. Determination of Dynamic Rheological Properties

Dynamic rheological properties of high-gluten wheat flour with different levels of OPE were evaluated following the meth-od outlined by Zhang et al. [20] using a rheometer (KinexusPro+, Malvern Instruments Ltd., UK). The dough was prepared by mixing with a farinograph for 5 minutes, followed by 5 minutes of mixing with paraffin-coated plate edges to minimize water loss. The dough was then allowed to rest at 25 °C for 30 minutes. The linear viscoelastic region of the sample was determined through strain scanning at 25 °C and 1 Hz. Both the storage modulus (G') and the viscous modulus (G'') were measured. The loss factor (tanδ) was calculated as the ratio of G'' to G'.

*S2.3. Bread Quality Analysis*

S2.3.1. Preparation of Bread

120g of high-gluten wheat flour with different contents of OPE (0.25%, 0.5%, 0.75%, 1%, g/g), 24g of sugar, 2g of yeast, 6.8g of butter, 1.2g of salt, and 73g of water were used to make the bread dough. Knead to create a smooth dough after combining the ingredients listed above. After 20 minutes of the first rise in the oven, the dough was removed to be kneaded and reshaped. Then it was left in the oven for 50 minutes for the second rise. After rising, the dough was put on a baking tray and baked at 175 °C for 20 minutes on both the higher and lower heats. The breads were then allowed to cool to room temperature before being put in self-sealing bags for the upcoming research [17].

S2.3.2. Determination of Total Phenolic Content, Total Flavonoid Content, and Antioxidant Activity of Crumbs

Preparation of polyphenol extract from crumbs: To create freeze-dried bread powder, the crumbs were vacuum-freeze-dried for 48 hours, then crushed and sieved through a 40-mesh screen. 40 mL of a 70% ethanol solution was combined with 2 g of the powder, and the mixture was let to soak for two hours. After 15 minutes of centrifuging the mixture at 3,500 g, the supernatant was extracted for additional examina-tion. The total phenolic content, total flavonoid content, and antioxidant activity of this supernatant were evaluated [26].

The total phenolic content was determined following the method out-lined by Sagar and Pareek [27] with minor changes. 0.15 mL of extract was mixed with a 10-fold Folin-Ciocalteu's reagent (1.5 mL, 0.2 moL/L). After extract was incubated for 3 min, sodium carbonate solution (10 mL, 7%) was added and incubated for 1 h away from light. Absorbance was read using spectrophotometer (UPG-722, Beijing Yotubook General Technology Co., Ltd.) at 785 nm. The results were expressed as mg gallic acid equivalents (GAE)/g DW.

Total flavonoid content was measured according to Sagar and Pareek [27] with minor modifications. Extracts (0.5 mL) were taken into test tubes and 1.5 mL sodium nitrate solution (1.6%, m/v) was poured into tubes. Then, aluminium chloride (3.2%, m/v) was poured in 250 μL quantity. Sodium hydroxide solution ( 8%, m/v) was poured and incubated at room temperature (28 °C) for 15 min. The absorbance was taken via spectrophotometer at 510 nm. The results were expressed as mg rutin equivalents (RE)/g DW.

Two techniques, such as the DPPH and ABTS free radical scavenging rate, were employed to quantify the antioxidant activity of the samples in order to increase its precision and predictability. With minor adjustments, the extract' ABTS free radical scavenging rate was determined using Sagar and Pareek et al.'s methodology [27]. Potassium persulfate (2.6 mmol/L) and ABTS (7.4 mmol/LM) solution were taken in 20 mL concentration each and left at room temperature for 12 h in the dark. Mix 50 μL of extract with 450 mL of ethanol and add 500 ml of ABTS mixture. Absorbance was measured at 723 nm. The DPPH free radical scavenging rate was determined according to the method of Sagar and Pareek et al [22]. Extract (2 mL) and 2 mL DPPH (0.4 mM) were mixed followed by incubation in dark for 0.5 h. Then, absorbance was measured at 517 nm via spectrophotometer. The DPPH and ABTS free radical scavenging rate was calculated as follows:

$$\text{DPPH/ABTS free radical scavenging rate (\%)} = \frac{\text{Acontrol} - \text{Asample}}{\text{Acontrol}}$$

S2.3.3. Color of Crumbs

The color parameters of the samples, including L* (lightness), a* (red/green), and b* (yellow/blue), were measured using a chromometer (Shenzhen Linshang Technology Co., Ltd., China), following the methodology outlined by Zhang et al.[20]. These values were obtained based on the CIE (Commission Internationale de l'Éclairage) color scale. The measurement process was performed under standard illuminant conditions, and the observer angle used was 10°, as recommended by the CIE.

S2.3.4. Specific Volume of the Bread

According to Wang et al. [16], the volume of bread was calculated by replacing the millet. After filling a 500 mL graduated cylinder, 500 mL of millet was emptied out and placed aside. To make sure the bottom of the 500 mL graduated cylinder was fully covered, a layer of the millet was added. The cooled bread was then put into the cylinder. The cylinder was then filled with millet until it reached the 500 mL calibration level. A 100 mL graduated cylinder was used to measure the leftover millet, and the volume was noted. Since the millet fills the area left by the bread in the 500 mL cylinder, this volume equals essentially the

bread's volume. The bread was then weighed using an electronic balance, and the mass of the bread was recorded. Specific volume ($cm^3/g$) was determined using bread volume divided by weight.

S2.3.5. Textural Characteristics of Bread

Texture measurements of bread were based on the method reported by Wang et al.[16] with minor modifications. Bread (15 mm × 15 mm × 20 mm) was placed on the carrier table of the food physical property analyzer (TA-TX PlusC, Stable Micro System, UK) in order to measure the samples' hardness (N), cohesiveness, springiness (mm), and chewiness (N). The property analyzer used a 36 mm cylindrical plastic probe, with a probe rise height of 40 mm, drop speed of 60 mm/s, and a compression ratio of 50%.

S2.2.6. Microstructure

In accordance with Tang et al.'s approach [28], 2 cm × 2 cm × 2 cm cube-shaped crumbs were fixed with 3% glutaraldehyde and freeze-dried. The samples underwent a gradient elution with 30%, 50%, 70%, 90%, and 100% ethanol following a rinse with 0.1 mol/L phosphate buffer. A cold-field SEM (Gemini 300, Carl Zeiss AG, Germany) operating at 20.0 KV was used to view the cross-sections of the freeze-dried samples after they had been adhered to the sample stage using conductive glue and sprayed with gold for 90 seconds. Photographs and observations were taken at 60x magnification.

S2.3.7. Sensory Evaluation of Bread

The sensory ratings of bread were evaluated using a nine-point hedonic scale, which includes color, flavor, texture, taste, and overall acceptability (1 being severely disliked and 9 being extremely liked), in accordance with the methodology of Sagar & Pareek [27]. The study involved thirty panelists. A random three-digit number was assigned to each sample, which was then arranged on a white plate and presented to the panelists in a random order. To reduce the influence of oral residues on the evaluation, the panelists were instructed to rinse their mouths with warm water before to sampling.

*S2.4. Data Analysis*

The findings are shown as mean ± standard deviation (SD), and each experiment was run in triplicate. To analyze the data, SPSS software, version 20.0, was used. In this study, the analysis of variances (ANOVA) procedure was used. Additionally, Duncan's multiple range tests were used to see whether there was a significant difference in the means of the various treatments. Consequently, a statistically significant difference was indicated by $p < 0.05$.

Statement of approval for the experiment: Every experiment was carried out in compliance with the applicable rules and regulations. The academic review commissions of Qiqihar University's College of Food and Bioengineering approved all experimental protocols prior to analysis.

Ethical statement: Prior to analysis, the academic review commissions of Qiqihar University's College of Food and Bioengineering examined and authorized the sensory evaluation panelists with written informed permission (Project Number: QH20241210, approved on 10 December 2024).

All participants signed an informed consent form after being fully informed about the study.
